# Supplementary material for: Iron’s role in soil organic carbon (de)stabilization in mangroves under land use change
Source: Nat Commun. 2024 Nov 30;15:10433. doi: 10.1038/s41467-024-54447-z (PMC11608346; doi:10.1038/s41467-024-54447-z)
Supplement: Supplementary file 1 — Supplementary Information [file 41467_2024_54447_MOESM1_ESM.pdf]

Supplementary material for

Iron's role in soil organic carbon (de)stabilization in mangroves under land use change

Francisco Ruiz [1*], Angelo Fraga Bernardino [2], Hermano Melo Queiroz [3], Xosé Luis Otero [4,5], Cornelia Rumpel [6] Tiago Osório Ferreira [1,7,8*]

[1] Department of Soil Science, "Luiz de Queiroz" College of Agriculture/University of São Paulo (ESALQ/USP), Av. Pádua Dias 11, Piracicaba, São Paulo 13418-900, Brazil

[2] Department of Oceanography, Federal University of Espírito Santo, Vitória, Espírito Santo, Brazil

[3] Department of Geography, University of São Paulo, Av. Prof. Lineu Prestes, 338, Cidade Universitária, 05508-900, São Paulo, SP, Brazil

[4] CRETUS, Department of Edaphology and Agricultural Chemistry, Faculty of Biology, University of Santiago de Compostela – USC, Rúa Lope Gómez de Marzoa, s/n. Campus sur, 15782, Santiago de Compostela, Spain

[5] REBUSC Network of Biological Stations of the University of Santiago de Compostela, A Graña Marine Biology Station, Ferrol, Spain

[6] CNRS, Institute for Ecology and Ecological Sciences, (iEES Paris, UMR Sorbonne Université, CNRS, INRAE, UPEC, IRD), Paris, France

[7] Center for Carbon Research in Tropical Agriculture (CCARBON) - University of São Paulo, Piracicaba, São Paulo, Brazil

[8] University of São Paulo, Research Centre for Greenhouse Gas Innovation – RCGI, Av. Professor Mello Moraes, 2231, São Paulo, SP, Brazil.

* Corresponding authors: francisco.ruiz@usp.br; toferreira@usp.br

Table S1. Parameters obtained after the sequential wet-chemical experiment using the MAOC fraction of a pristine mangrove. The Fe concentrations and OC:Fe ratios refer to Fe and OC extracted within each step (cit, asc and cbd). Other parameters (ED, DSC- T_{50} and $CH:COO^-$ and C:N ratios) were measured in the sample before extraction and in the residue after each extraction. Refer to Fig. S1 for a visual representation of the extraction processes.

Depth	Treatment	Fe	OC:Fe ratio	ED	DSC-T_{50}	$CH:COO^-$ ratio	C:N ratio
cm		g kg $^{-1}$	mol mol^{-1}	J mg^{-1}C	°C		
0-10	**Before extraction**			3.3	329.8	1.7	11.9
	**cit**	4.31 ± 0.08	4.0 ± 0.1	3.7	343.6	2.5	12.1
	**asc**	1.14 ± 0.02	12.5 ± 0.0	4.0	352.3	4.0	15.1
	**cbd**	1.21 ± 0.08	4.7 ± 1.8	3.7	347.9	3.0	16.5
40-50	**Before extraction**			3.3	330.8	1.7	13.7
	**cit**	5.16 ± 0.11	1.5 ± 0.5	3.9	344.5	3.6	15.1
	**asc**	1.44 ± 0.03	13.4 ± 2.2	4.2	352.5	5.3	18.1
	**cbd**	0.45 ± 0.05	4.6 ± 7.1	4.0	340.0	2.8	17.7
80-100	**Before extraction**			3.9	337.6	1.7	13.8
	**cit**	3.37 ± 0.04	2.9 ± 0.3	4.6	348.8	3.2	15.7
	**asc**	0.57 ± 0.01	10.4 ± 0.3	4.5	354.1	4.5	23.6
	**cbd**	1.97 ± 0.17	4.0 ± 0.5	4.1	338.2	4.1	21.6

ED: Energy density; DSC-T_{50}: the temperature at which half of the exothermic energy is released; cit: Na-citrate pH 6; asc: Citrate-ascorbate pH 6; cbd: citrate bicarbonate dithionite.

Table S2. Results from adsorption and co-precipitation experiments of humic acid (HA) with different Fe phases and molar C:Fe ratios

Fe phase / initial molar C:Fe ratio	mg HA g^{-1} solid
Adsorption	
Ferrihydrite	65.3 ± 2.0
Goethite	26.7 ± 0.4
Co-precipitation	
C:Fe = 12	664.5 ± 11.3
C:Fe = 24	720.2 ± 15.4

Methods referring to Table S2: Adsorption and co-precipitation experiments

Ferrihydrite was synthesized using the "solvent-deficient" method by mixing $Fe(NO_3)_3 \cdot 9H_2O$(s) and NH_4HCO_3(s) in a 1:3 molar ratio with a mortar and pestle [1]. The resulting slurry containing ferrihydrite was dried at 80°C and rinsed five times with 70% ethanol. Goethite and humic acid (HA) were obtained from Sigma-Aldrich. Batch experiments were conducted to study the adsorption of HA onto the Fe oxides. A stock HA solution was prepared by dissolving solid HA in 10 mM NaOH, followed by pH adjustment to 6 using 1M HCl. For the adsorption experiments, the iron oxides were suspended in deionized water and the pH was adjusted to 6.0. The suspensions and the HA were placed into 50 ml centrifuge tubes. The final solid concentration was 2g L^{-1} and the HA concentration was 360 mg L^{-1}. The suspension was shaken for 24 h at 150 rpm. The suspensions were then centrifuged, and the supernatants were collected. The HA concentration (360 mg L^{-1}) was chosen in excess to saturate the adsorption sites, based on previous studies [2,3].

Co-precipitates of Al and Fe oxides with HA were also prepared following the method of Chen et al. (2014). In this procedure, 0.2 g of $Fe(NO_3)_3 \cdot 9H_2O$ was stirred with 200 ml of HA solutions (360 and 720 mg C L^{-1}) to generate initial molar C:Fe ratios of 12 and 24. The pH of the suspensions was gradually raised to 7 by adding 0.1 M NaOH. The resulting precipitates were centrifuged and rinsed once with deionized water to remove any excess HA solution.

The amount of HA adsorbed or co-precipitated was quantified using UV-Vis analysis at 600 nm, with a quartz cell [4]. Calibration curves for HA were generated for concentrations ranging from 4 to 360 mg L^{-1} (R^2 > 0.999). The amount of HA was determined by comparing the HA concentrations in solution before and after the adsorption or co-precipitation processes. All treatments were prepared in duplicates.

Table S3. Comparison of wet chemical extractions in freeze-dried and moist soil samples from different sites and depths. Values represent concentrations, in mg kg^{-1}, of iron extracted by citrate (cit), citrate-ascorbate (cit-asc), and citrate-bicarbonate-dithionite (cbd) extractions. Data are presented as mean ± standard deviation.

Site	Depth (cm)	cit		cit-asc		cbd	
		freeze-dried	moist	freeze-dried	moist	freeze-dried	moist
		---mg kg^{-1}---					
Mangrove (pristine)	0-10	2.58±0.13	1.99±0.09	0.85±0.04	0.62±0.03	0.37±0.02	0.23±0.01
	40-50	4.21±0.2	3.92±0.2	0.92±0.04	0.99±0.04	0.33±0.02	0.28±0.01
	80-100	3.12±0.12	3.32±0.07	0.84±0.03	1.75±0.29	0.3±0.01	0.34±0.05
Mangrove (shrimp pond)	0-10	0.88±0.03	0.84±0.04	0.59±0.02	0.69±0.04	0.14±0.01	0.13±0
	40-50	0.83±0.03	0.91±0.05	0.21±0.01	0.32±0.02	0.07±0	0.08±0
	80-100	0.8±0.04	0.73±0.04	0.22±0.01	0.2±0.01	0.04±0	0.04±0
Shrimp pond	0-10	0.21±0.01	0.21±0.01	0.27±0.01	0.24±0.01	0.36±0.02	0.33±0.01
	40-50	0.33±0.02	0.25±0.01	0.18±0.01	0.16±0.01	0.13±0	0.11±0
	80-100	0.44±0.02	0.65±0.03	0.25±0.01	0.36±0.02	0.05±0	0.07±0
Pasture	0-10	0.03±0	0.04±0.01	0.05±0	0.11±0.01	0.07±0	0.14±0.01
	40-50	0.01±0	0.02±0	0.01±0	0.01±0	0.02±0	0.02±0
	80-100	0±0	0.01±0	0±0	0.02±0	0.01±0	0.02±0

Table S4. Comparison of wet chemical extractions in freeze-dried and moist soil samples from different sites and depths. Values represent the relative distribution (%) of iron extracted by citrate (cit), citrate-ascorbate (cit-asc), and citrate-bicarbonate-dithionite (cbd) extractions.

Site	Depth	cit		cit-asc		cbd	
		freeze-dried	moist	freeze-dried	moist	freeze-dried	moist
Mangrove (pristine)	0-10	68	70	22	22	10	8
	40-50	77	76	17	19	6	5
	80-100	73	61	20	32	7	6
Mangrove (shrimp pond)	0-10	55	51	36	42	9	8
	40-50	75	70	19	24	6	6
	80-100	75	75	21	21	4	4
Shrimp	0-10	25	27	32	31	43	42
	40-50	52	49	28	30	20	21
	80-100	59	60	34	33	7	7
Pasture	0-10	18	13	36	40	46	48
	40-50	33	37	31	25	37	38
	80-100	30	14	29	46	41	40

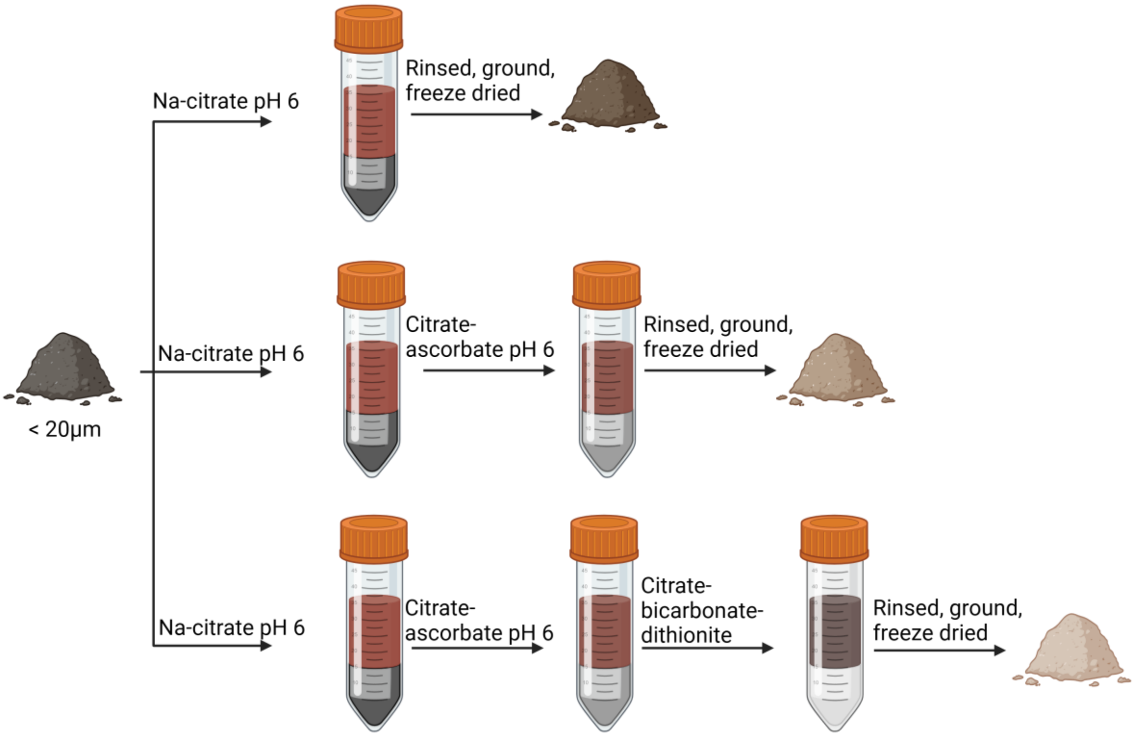

Fig S1. Illustration of the experimental scheme involving wet-chemical extractions. Subsamples from the MAOC fraction of a pristine mangrove, at depths of 0-10, 40-50 and 80-100 cm, in duplicate, underwent distinct sequences of chemical extractions. Following these chemical treatments, the subsamples were then analyzed for total organic carbon (TOC), as well as through infrared and thermal analysis. Created in BioRender. Gomes Viana, D. (2024) https://BioRender.com/r77i059.

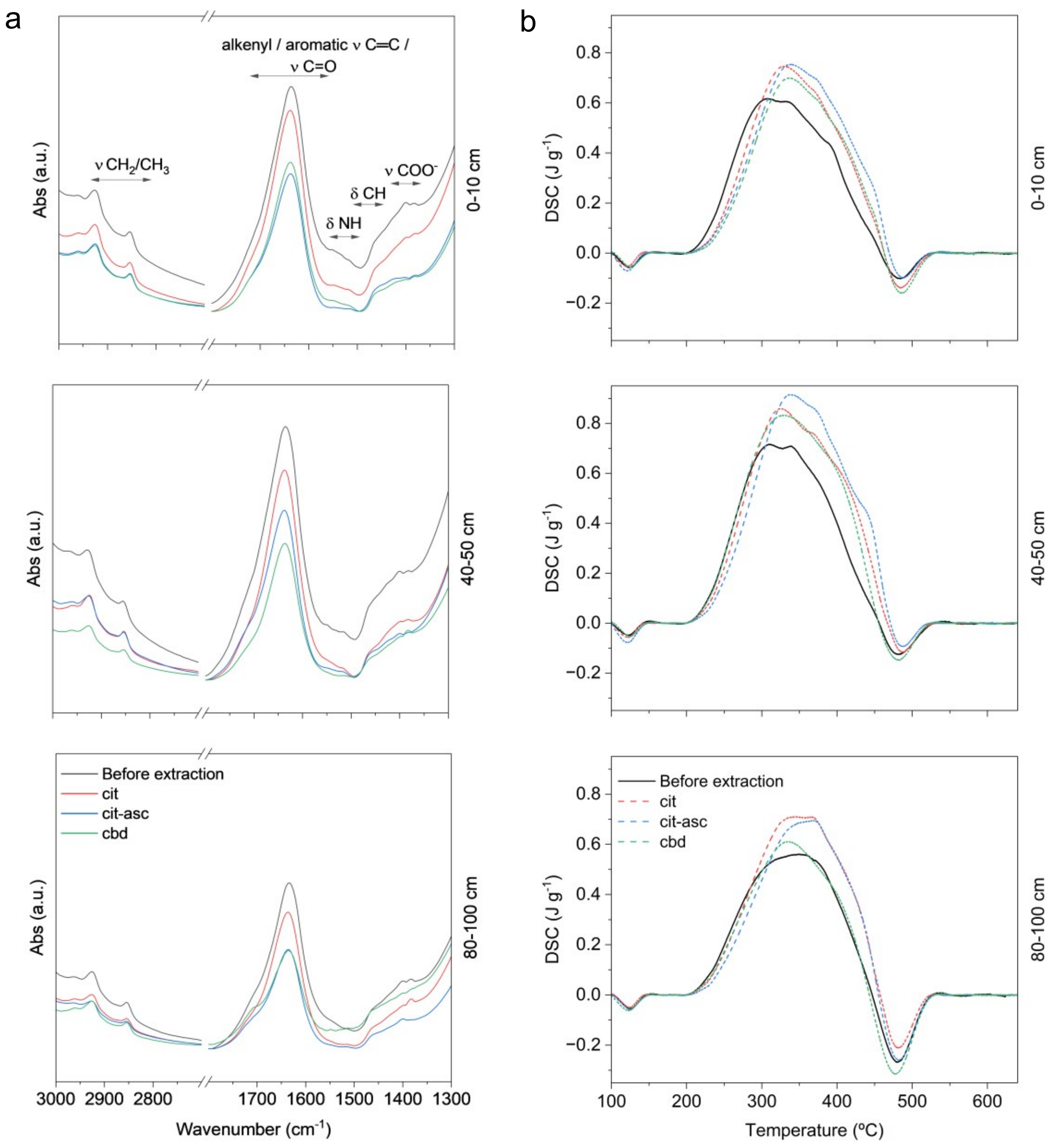

Fig S2. Samples from a pristine mangrove containing the MAOC fraction before and after the Fe sequential extraction. **a** FTIR spectra **b** DSC thermograms (cit: Na-citrate pH 6; asc: Citrate-ascorbate pH 6; cbd: Citrate bicarbonate dithionite).

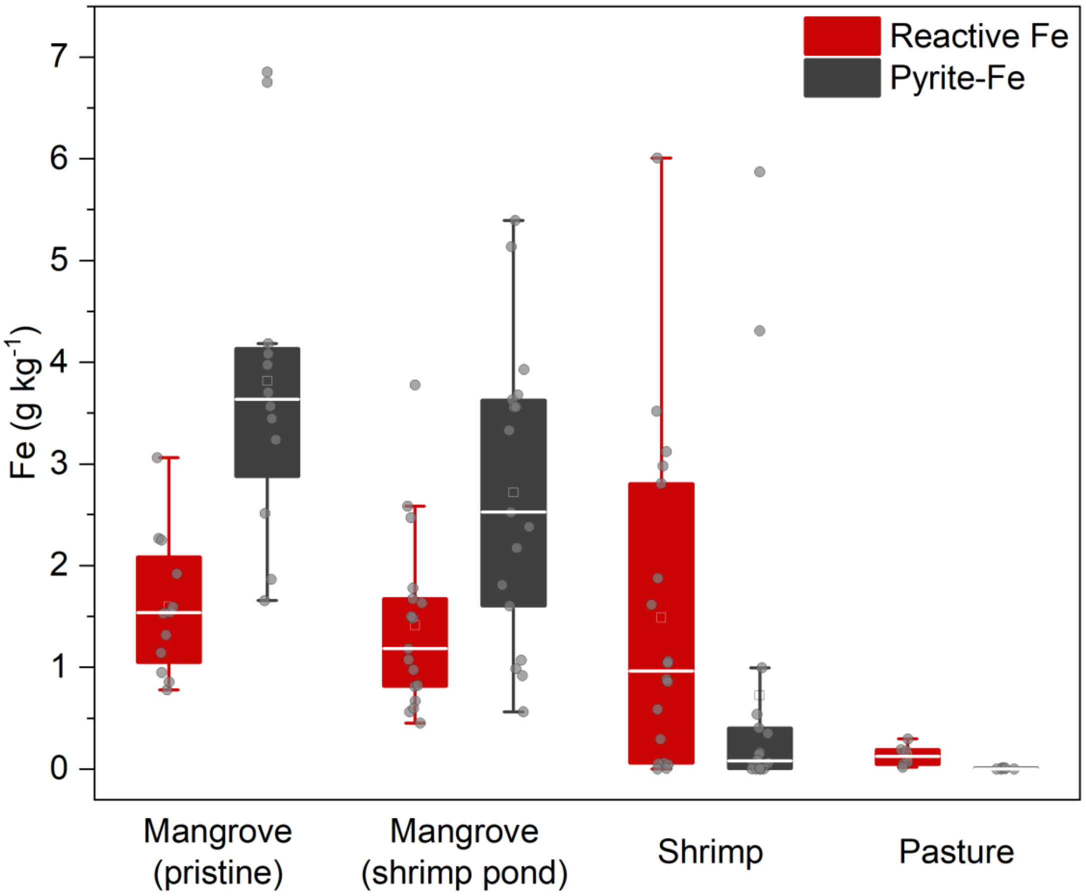

Fig S3. Concentrations of reactive and pyritic Fe in soils across different land-uses. The box represents the interquartile range (IQR), with the horizontal line showing the median, the square indicating the mean and whiskers extending to the maximum and minimum values within 1.5 times the IQR. Points beyond the whiskers indicate outliers.

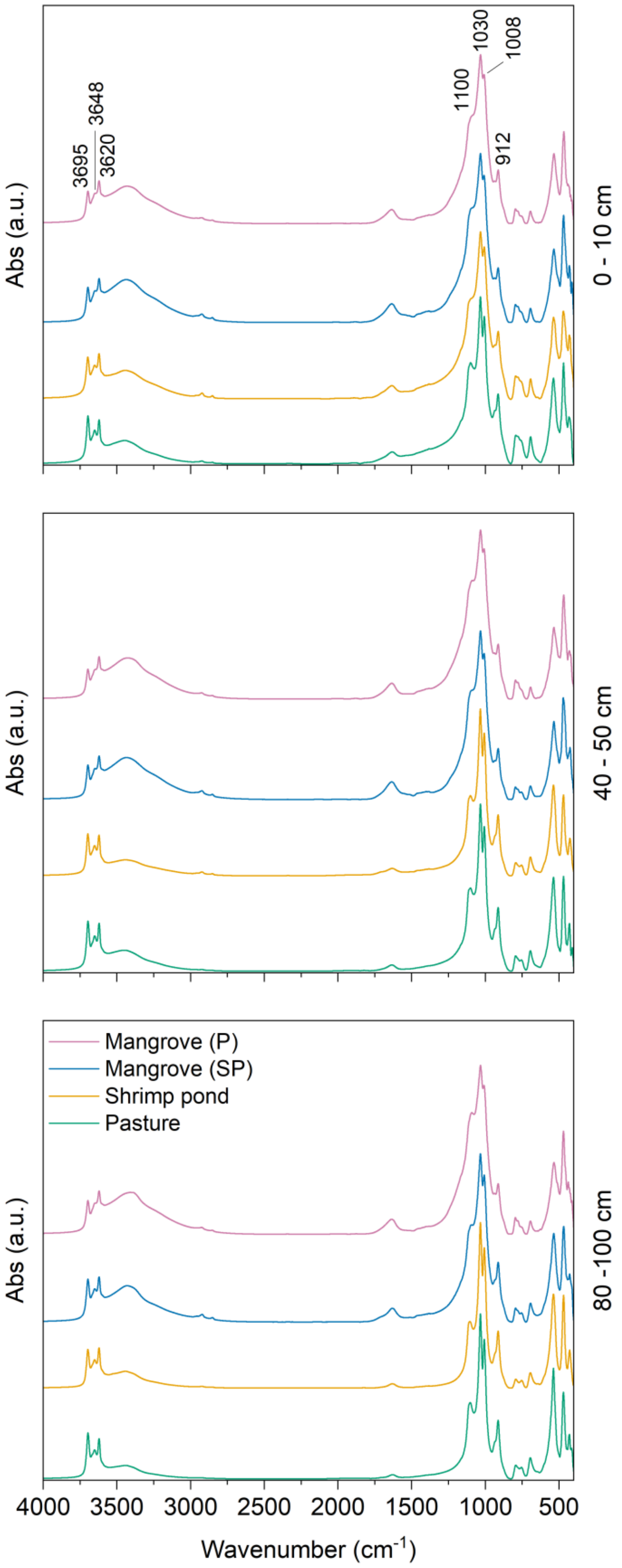

Fig .S4 FTIR spectra obtained from the MAOC of the studied soils. Peaks at 3695, 3648, 3620, 1100, 1030, 1008 and 912 cm^{-1} are typical of kaolinite [5].

Supplementary References

1. Smith, S. J. *et al.* Novel Synthesis and Structural Analysis of Ferrihydrite. *Inorg Chem* 51, 6421–6424 (2012).
2. Gu, B., Schmitt, J., Chen, Z., Liang, L. & McCarthy, J. F. Adsorption and desorption of different organic matter fractions on iron oxide. *Geochim Cosmochim Acta* 59, 219–229 (1995).
3. Chen, C., Dynes, J. J., Wang, J. & Sparks, D. L. Properties of Fe-Organic Matter Associations via Coprecipitation versus Adsorption. *Environ Sci Technol* 48, 13751–13759 (2014).
4. Li, P. & Hur, J. Utilization of UV-Vis spectroscopy and related data analyses for dissolved organic matter (DOM) studies: A review. *Crit Rev Environ Sci Technol* 47, 131–154 (2017).
5. Chukanov, N. V. *Infrared Spectra of Mineral Species: Extended Library*. (Springer Science & Business Media, 2013).
